# Cognitive behavioral therapy for the treatment of PTSD

**DOI:** 10.1080/20008198.2017.1351219

**Published:** 2017-09-29

**Authors:** Ioannis Syros

**Affiliations:** ^a^ Department of Child Psychiatry, Medical School, National and Kapodistrian University of Athens, “Aghia Sophia” Children’s Hospital, Athens, Greece

**Keywords:** Children and adolescents, trauma, post-traumatic stress disorder (PTSD), cognitive behavioral therapy, cognitive behavioral intervention on trauma in schools

## Abstract

**Background**: A variety of therapeutic approaches have been implemented to treat post-traumatic stress symptoms and concomitant difficulties. Those interventions focusing on trauma are considered first-line treatment for children and adolescents. Trauma Focused Cognitive Behavioral Therapy (TF-CBT, Cohen, Mannarino & Deblinger, 2006), and a similar type of group intervention, the Cognitive Behavioral Intervention on Trauma in Schools (CBITS, Stein et al., 2003), have received the most empirical support through randomized controlled trials (RCTs).

TF-CBT is designed for children with PTSD with or without comorbid depression, anxiety, and other emotional problems associated with trauma (shame, self-immolation). It is delivered separately to children and their parents in 12–20 sessions and is suitable for youth aged 3 to 18 years. It is also provided as a group. An important part of the intervention is gradual exposure to those stimuli that are feared and avoided by the child.

The usual elements of the TF-CBT could be described with the acronym PRACTICE, more specifically: (1) Psychoeducation. (2) Parenting skills. (3) Relaxation skills. (4) Affective modulation skills. (5) Cognitive coping and processing through narrative trauma. (6) In vivo control (mastery) of the trauma reminders. (7) Combined child and parent sessions. (8) Strengthening future security and development.

**Objective**: This review provides an overview of two CBT protocols, TF-CBT and CBITS. It also presents several other promising CBT protocols.

**Method**: Systematic searches were conducted of all relevant bibliographic databases using the following terms: child, adolescent, trauma, posttraumatic stress, cognitive behavioral therapy, randomized control trials. The search covered the period from 1996 to 2015 and was conducted in May 2017.

**Results**: TF-CBT is studied in 10 RCTs, involving a total of more than 900 children, demonstrating clinically significant improvement. Follow-up studies show the sustainability of benefits for 6 months, 1 year and 2 years after treatment.

As for CBITS, only one RCT was found, plus one quasi-experimental and four field trials. It is the best-researched CBT protocol in a child-care group and includes all elements of PRACTICE, with the exception of the parent component, which is limited and optional. The component is predominantly conducted during ‘short break sessions’ in which each child meets their primary group therapist. CBITS has been applied to two major studies in children exposed to violence in the community. In the study by Stein et al. (2003), CBITS was superior to the waiting list in reducing PTSD and depression cases.

Several other CBT protocols have been found, embodying promising practices that could facilitate the application of CBT to special populations of different origins and specificities in symptomatology. For example, UCLA Τrauma and Grief Component Therapy has been primarily provided in schools, showing a benefit to teenagers exposed to war, community violence or even terrorist acts. Prolonged exposure therapy showed therapeutic benefit, although it was provided by staff not trained in exposure therapy, while in SPARCS and TARGET–A protocols gradual exposure to trauma was not a core component of the treatment. Finally, the nine-session protocol STEPS, applied to teenage girls who had experienced single rape, showed improvement mainly in chronic symptoms (Bicanic, De Roos, Van Wesel, Sinnema, & Van, 2014).

**Conclusions**: Effective treatments for empirically supported PTSD are available, most of which include CB approaches. CB therapy and psychoeducation can provide structure and support when anxiety and avoidance discourage trauma exploration, while therapeutic gains seem to be maintained over time. Future directions characterize (a) research on promising practices and the potential of new interventions, (b) the exploration of providing interventions to specific cultural populations, as well as (c) the further study of those elements that ‘work’ in CB treatments, and their importance in empowering new health professionals trained in these approaches.
